# Genome sequence of bacteriophage Djungelskog isolated from an *Arthrobacter globiformis* culture

**DOI:** 10.1128/mra.01294-23

**Published:** 2024-02-20

**Authors:** Abigail M. Oliveros, Shelby A. McDougall, Miles A. Snyder, Sara K. Snowden, Joseph D. Richard, Christopher M. Rao, Marybeth Ponce, Christopher J. Pitonza, Mira Ozcelik, Sofia S. Mannina, Juliana R. Magna, Andrew S. Lopez, Linnea C. Gustafson, Brynn K. Glackin, Abigail E. Dolge, Nate D. DeLancy, Andrew B. C. Davis, Thomas P. Davis, Max Blagodar, Sydney N. Natale, Megan K. Dennis, Elizabeth A. Godin

**Affiliations:** 1 Department of Biology, Marist College, Poughkeepsie, New York, USA; DOE Joint Genome Institute, California, USA

**Keywords:** bacteriophages, phages

## Abstract

Actinobacteriophage Djungelskog was isolated from a sample of degraded organic material in Poughkeepsie, NY, using *Arthrobacter globiformis B-2979*. Its genome is 54,512 bp and encodes 86 putative protein-coding genes. Djungelskog has a siphovirus morphology and is assigned to cluster AW based on gene content similarity to actinobacteriophages.

## ANNOUNCEMENT

Bacteriophages are increasingly relevant in the field of biotechnology, agriculture, and medicine, particularly for controlling bacterial growth (1). Here, we report on bacteriophage Djungelskog that infects *Arthrobacter*, the latter capable of breaking down complex hydrocarbons and is therefore a potential use in bioremediation (2).

**Fig 1:**
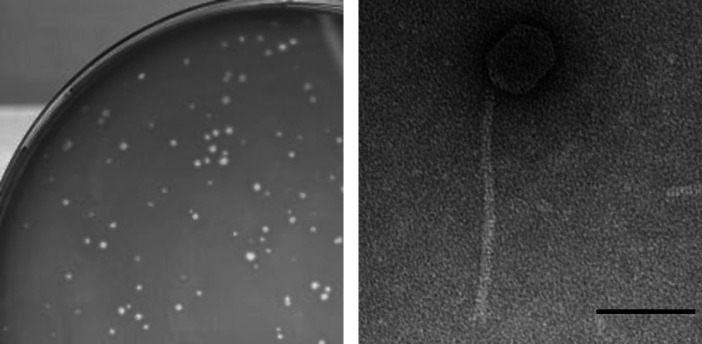
Characterization of Actinobacteriophage Djungelskog. Image of plaques that were clear and approximately 0.7 mm in diameter (left) and transmission electron micrograph (right) of Djungelskog lysate negatively stained with UranylLess on a 300-mesh copper grid and imaged with a Hitachi HT7800 120 kV transmission electron microscope (scale bar = 100 nm).

In August 2022, Djungelskog was isolated directly from an environmental sample consisting of degraded leaves and dirt, from Poughkeepsie, NY (GPS: 41.722735 N, 73.92931 W), using standard procedures (3). The sample was isolated in peptone-yeast calcium (PYCa) liquid media, then filtered through a 0.22-µm filter. The filtered sample was plated in PYCa top agar with *Arthrobacter globiformis B-2979* and incubated at 30℃ for 48 h, yielding clear plaques that were ~0.7 mm in diameter (Fig. 1) after three rounds of purification. Using negative staining transmission electron microscopy, a siphovirus morphology with a prolate capsid (72–73 nm in length, 53–58 nm in width) and tail (length of 237–244 nm) was observed (*n* = 9, Fig. 1) (4).

Genomic DNA for Djungelskog was isolated from lysate using Promega Wizard DNA Clean-Up Kit, prepared for sequencing using NEB Ultra II Library Kit, and sequenced using Illumina MiSeq (v3 reagents), to yield 471,444,150-base single-end reads. The reads were assembled into a contig with 1,241× coverage using Newbler v2.9 and checked for accuracy using Consed v29 (5, 6). Default parameters were used for all software, unless otherwise stated. The resulting genome is 54,512 bp with a GC% of 51.70% and a 3′ single-stranded genome end (CGCCGACCT).

Djungelskog’s genome was automatically annotated using DNA Master v5.23.6 (https://phagesdb.org/DNAMaster/), embedded with Genemark v2.5p (7) and Glimmer v3.02 (8). The annotations were refined using Starterator v519 and Phamerator v528 (9) for comparison with similar phages. BLASTp (10) searches against the NCBI non-redundant and actinobacteriophage databases and HHPred (11) searches against the PDB_mmCIF70, Pfam-A, UniProt-SwissProt-viral70, and NCBI_Conserved_Domains (CD) databases were used to assign putative functions. Using ARAGORN (12) and tRNA scan-SE (13), it was found that this genome did not contain any tRNAs. SOSUI (14) and TMHMM (15) revealed six proteins with potential transmembrane regions. The annotation process revealed 86 genes, of which 21 genes could be assigned putative functions. Based on gene content similarity (GCS) of >35% to sequenced actinobacteriophages, using the GCS tool at the Actinobacteriophages database (https://phagesdb.org/), Djungelskog was assigned to the AW cluster (16, 17).

As with other cluster AW genomes, all genes are transcribed in the same direction, with virion structure and assembly genes clustered across one half of the genome and DNA metabolism genes clustered across the other half. Djungelskog encodes for a major capsid and protease protein, homologs of which are found in actinobacteriophage of various clusters and isolated on different bacteria, including cluster B isolated on *Mycobacteria*, cluster CC isolated on *Rhodoccocus*, cluster DJ isolated on *Gordonia*, cluster EL isolated on *Microbacteria*, and clusters AM, AU, FI, and FK isolated on *Arthrobacter*. No immunity repressor or integrase functions were identified, and clear plaque phenotype was observed, suggesting a lytic lifecycle.

## Data Availability

Djungelskog is available at GenBank with Accession No. OR521078 and Sequence Read Archive (SRA) No. SRX20165789.
